# Neuropsychiatric symptoms associated with the COVID-19 and its potential nervous system infection mechanism: the role of imaging in the study

**DOI:** 10.1093/psyrad/kkab019

**Published:** 2021-12-22

**Authors:** Yanyao Du, Wei Zhao, Lei Du, Jun Liu

**Affiliations:** Department of Radiology, Second Xiangya Hospital of Central South University, Changsha 410011, Hunan Province, China; Department of Radiology, Second Xiangya Hospital of Central South University, Changsha 410011, Hunan Province, China; Clinical Research Center for Medical Imaging in Hunan Province, Changsha 410011, Hunan, China; Department of Psychiatry and Behavioral Neuroscience, University of Cincinnati College of Medicine, Cincinnati 45255, OH, USA; Department of Radiology, Second Xiangya Hospital of Central South University, Changsha 410011, Hunan Province, China; Clinical Research Center for Medical Imaging in Hunan Province, Changsha 410011, Hunan, China; Department of Radiology Quality Control Center, Hunan Province, Changsha 410011, Hunan, China

**Keywords:** COVID-19, SARS-CoV-2, nervous system, imaging sequences, pathogenesis, neuropsychiatric sequelae, brain function alteration

## Abstract

The epidemic of coronavirus disease 2019 (COVID-19) has broken the normal spread mode of respiratory viruses, namely, mainly spread in winter, resulting in over 230 million confirmed cases of COVID-19. Many studies have shown that severe acute respiratory syndrome coronavirus-2 (SARS-CoV-2) can affect the nervous system by varying degrees. In this review, we look at the acute neuropsychiatric impacts of COVID-19 patients, including acute ischemic stroke, encephalitis, acute necrotizing encephalopathy, dysosmia, and epilepsy, as well as the long-term neuropsychiatric sequelae of COVID-19 survivors: mental disorder and neurodegenerative diseases. In particular, this review discusses long-term changes in brain structure and function associated with COVID-19 infection. We believe that the traditional imaging sequences are important in the acute phase, while the nontraditional imaging sequences are more meaningful for the detection of long-term neuropsychiatric sequelae. These long-term follow-up changes in structure and function may also help us understand the causes of neuropsychiatric symptoms in COVID-19 survivors. Finally, we review previous studies and discuss some potential mechanisms of SARS-CoV-2 infection in the nervous system. Continuous focus on neuropsychiatric sequelae and a comprehensive understanding of the long-term impacts of the virus to the nervous system is significant for formulating effective sequelae prevention and management strategies, and may provide important clues for nervous system damage in future public health crises.

## Introduction

The epidemic of coronavirus disease 2019 (COVID-19) has broken the normal spread mode of respiratory viruses: the severe acute respiratory syndrome coronavirus-2 (SARS-CoV-2) spread not only in the winter of 2019 and 2020 but also in the summer of 2020 and 2021 (Wang *et al*., 2020a). According to the WHO Coronavirus (COVID-19) Dashboard (https://covid19.who.int/), there have been over 230 million confirmed cases of COVID-19 worldwide, as of 28 September 2021. Although >5.9 billion people have been vaccinated against the COVID-19, the epidemic has not been effectively controlled in many parts of the world.

The coronavirus was discovered by Fred Beaudette and Charles Hudson in 1937, and named by June Almeida in 1967. Because the spike glycoproteins on the virus particles under the electron microscope were like the crown, it was named “corona” in Arabic. So far, besides SARS-CoV-2, six coronaviruses that can infect humans have been discovered, including human coronaviruses (HCoV) OC43, 229E, NL63, and HKU1; severe acute respiratory syndrome coronavirus (SARS-CoV); and Middle-East respiratory syndrome coronavirus (MERS-CoV) (Drosten *et al*., 2003; Hamre and Procknow, 1966; Lau *et al*., 2006; Lia *et al*., 2004; McIntosh *et al*., 1967; Zaki *et al*., 2012; Zhou *et al*., 2020a). A detailed comparison of these seven coronaviruses is shown in Table 1.

**Table 1:** Characteristic of seven coronaviruses.

Characteristic	SARS-CoV-2	MERS-CoV	HCoV- HKU1	HCoV- NL63	SARS-CoV	HCoV- OC43	HCoV-229E
Occurrence time	December 2019	September 2012	January 2005	April 2004	April 2003	1967	1965
Genus	β-CoVs, lineage B	β-CoVs, lineage C	β-CoVs, lineage A	α-CoVs	β-CoVs, lineage B	β-CoVs, lineage A	α-CoVs
Binding receptor	ACE2	DPP4 (CD26)	__	ACE2	ACE2	9-O-acetylated sialic acid	Human aminopeptidase N (CD13)
Receptor distribution	Nervous, respiratory, cardiovascular, digestive, and urinary systems	Respiratory, digestive, and urogenital systems	Respiratory system	Digestive and urinary systems	Respiratory, digestive, and urinary systems	Respiratory and digestive systems	Respiratory and digestive systems
Severity of infection	Severe symptoms	Severe symptoms	Mild or moderate symptoms	Mild or moderate symptoms	Severe symptoms	Mild or moderate symptoms	Mild or moderate symptoms

Coronaviruses can be divided into four genera: α, β, γ, and δ, Among them, β-CoVs can be divided into four independent lineages A, B, C, and D. ACE2: angiotensin-conversion enzyme 2. DPP4: dipeptidyl peptidase 4 (also known as CD26).

The four HCoV strains (229E, NL63, OC43, and HKU1) are common coronaviruses that usually cause mild or moderate upper respiratory tract infections. Symptoms mainly include cough, rhinorrhea, headache, sore throat, fever, etc., which are common in children (Gaunt *et al*., 2010; Kuypers *et al*., 2007; Lau *et al*., 2006; Zeng *et al*., 2018). However, MERS-CoV, SARS-CoV, and SARS-CoV-2 not only can cause severe symptoms of the respiratory system (Booth *et al*., 2003; Mackay and Arden, 2015; Ozma *et al*., 2020; Peiris *et al*., 2003), but can also cause damage to other multiple systems, such as the urinary and cardiovascular systems (Amsalem *et al*., 2021; Mackay and Arden, 2015; Shi *et al*., 2020; Su *et al*., 2020; Yin and Wunderink, 2018). In addition, as the first symptom and sequelae of COVID-19, neuropsychiatric symptoms have been attracted more and more attention (AboTaleb, 2020; Mao *et al*., 2020; Paterson *et al*., 2020; Varatharaj *et al*., 2020). The detail mechanisms of virus infection in the brain or neuropsychiatric symptoms in acute and chronic stages are still unclear. Therefore, timely and continuous study of the development of neuropsychiatric symptoms in patients with COVID-19 has important clinical significance for disease monitoring and treatment strategy.

In this review, we reviewed previous studies related to MERS-CoV, SARS-CoV, and COVID-19 case reports, as well as the acute neuropsychiatric symptoms of COVID-19 patients, and the long-term neuropsychiatric sequelae of COVID-19 survivors may be caused by changes in brain function. Then, we discussed several potential mechanisms of SARS-CoV-2 infection of brain.

## Acute neuropsychiatric symptoms of COVID-19 patients

Imaging examinations, especially MRI, have advantages for nervous system diseases, but in the early stage of the epidemic, nervous system symptoms have not been attracted attention. In addition, strict isolation and preventive measures may limit many patients with mild neurological symptoms from receiving detailed imaging examination (Jain *et al*., 2020). Therefore, most of the symptoms in the acute stage of COVID-19 patients were reported by cases.

### Acute ischemic stroke

Stroke refers to a group of diseases with acute cerebral vascular circulation disorder, and the typical clinical manifestations are limb hemiplegia, aphasia, mental symptoms, vertigo, ataxia, choking cough, and even to coma and death in severe cases (Sacco *et al*., 2013). Acute ischemic stroke is currently reported as the most common cerebral vascular disease in patients with COVID-19 (Wang *et al*., 2020b), and is also the most common neurological manifestation associated with COVID-19 found in neuroimaging (Jain *et al*., 2020). The imaging findings of acute ischemic stroke related to COVID-19 were the same as those of localized ischemic stroke caused by other diseases.

Ischemic stroke often occurs 2 weeks after COVID-19 patients show neurological symptoms (Ellul *et al*., 2020a; Zhou *et al*., 2020b). Large retrospective studies in several countries have reported cases of acute ischemic stroke with different severities (Benussi *et al*., 2020; Jain *et al*., 2020; Klok *et al*., 2020; Lodigiani *et al*., 2020; Mao *et al*., 2020; Varatharaj *et al*., 2020). Detailed sample size and incidence rate of acute ischemic stroke are shown in Table 2. Through the studies in these five countries, we found that there were significant differences in the incidence of cerebral vascular circulation disorder reported by various countries, which may be related to the following two reasons: on the one hand, these six studies were retrospective studies, and there were deviations in data selection. More prospective studies are needed to verify this result in the future. On the other hand, we found two studies with low incidence rate of acute ischemic stroke. They used prophylactic thromboprophylaxis for all hospitalized patients or 100% intensive care patients and 75% of the general wards (Jain *et al*., 2020; Lodigiani *et al*., 2020). This finding may be of great significance to the formulation of treatment plan for hospitalized COVID-19 patients to prevent the occurrence of acute cerebral vascular circulation disorder.

**Table 2:** Summary of studies on COVID-19 patients with acute ischemic stroke.

Studies	Countries	Recruitment time	Sample size	Morbidity
(Mao *et al*., 2020)	China	16 January 2020 to 19 February 2020	214	5 (5.7%)
(Jain *et al*., 2020)	New York	1 March 2020 to 13 April 2020	3218	35 (1.1%)
(Varatharaj *et al*., 2020)	UK	2 April 2020 to 26 April 2020	153	57 (45.6%)
(Lodigiani *et al*., 2020)	Italian	13 February 2020 to 10 April 2020	388	9 (2.3%)
(Benussi *et al*., 2020)	Italian	21 February 2020 to 5 April 2020	173	43 (76.7%)
(Klok *et al*., 2020)	Netherlands	7 March 2020 to 5 April 2020	184	3 (1.6%)

Moreover, we also summarized six (21 COVID-19 patients) detailed case reports of acute ischemic stroke (Avula *et al*., 2020; Basi *et al*., 2020; Beyrouti *et al*., 2020; Diaz-Segarra *et al*., 2020; Oxley *et al*., 2020; Zhai *et al*., 2020), and found that the common symptoms were disorientation, hemiplegia, and dysarthria. In addition, young and elderly patients with COVID-19 may suffer from acute ischemic stroke, but most of them are older. At present, it is reported that the minimum age of onset of COVID-19 related acute ischemic stroke is 33 years old (Oxley *et al*., 2020), and most of them are over 60 years old.

Many potential causes of COVID-19 related acute ischemic stroke have been suggested, but the exact pathogenesis is unclear (Bhatia and Srivastava, 2020). Some scholars believe that the occurrence of acute cerebral vascular circulation disorders are not related to SARS-CoV-2 infection, because most of these patients have underlying diseases, including diabetes, hypertension, and cardiovascular diseases, which are known high risk factors for cerebrovascular diseases (Li *et al*., 2020a), so the time of cerebrovascular diseases during the infection of SARS-CoV-2 may be just an accident (Spence *et al*., 2020). However, we agree with most scholars that SARS-CoV-2 combined with vascular endothelial cells induces hypercoagulability, thereby activating the contact and complement system to cause neurological damage (Spence *et al*., 2020; Yaghi *et al*., 2020). There are two aspects of evidence to support this conjecture: first, acute ischemic stroke also occurs in some young patients without basic diseases (Oxley *et al*., 2020); second, in the cases with D-dimer level examination, the index increased significantly (Diaz-Segarra *et al*., 2020), and several studies have shown that high levels of D-dimer predict disease severity and hospitalized COVID-19 patients’ mortality (Li *et al*., 2020a; Yao *et al*., 2020; Yu *et al*., 2020; Zhang *et al*., 2020).

### Encephalitis

Encephalitis is an inflammatory process of the central nervous system, and is divided into three categories according to infection and the time of symptoms, including primary encephalitis, para-infection or postinfectious encephalitis, and autoimmune or paraneoplastic encephalitis (Achar and Ghosh, 2020). It is difficult to distinguish between primary encephalitis and postinfectious encephalitis caused by SARS-CoV-2. These two types of encephalitis were distinguished only by detecting cerebrospinal fluid (CSF) reverse transcriptase–polymerase chain reaction (RT–PCR), intrathecal synthesis of SARS-CoV-2 specific antibody, or by biopsy to detect whether there is SARS-CoV-2 antigen or RNA in brain tissue (Koralnik and Tyler, 2020). If any of these three tests is positive, it supports the diagnosis of primary encephalitis, inflammation caused by direct virus infection. The distinction between the first two categories and the third category also depends on laboratory examination. Autoimmune or paraneoplastic encephalitis can be excluded when CSF pleocytosis and protein elevation are detected (Ellul and Solomon, 2018). In addition, although electroencephalogram and imaging examinations (CT and MRI) may not distinguish the encephalitis, but they can show the damage of inflammation caused for various reasons to the nervous system.

So far, the symptoms of encephalitis in patients with COVID-19 were mostly reported by cases. We reviewed these case reports and divided them into the following three categories according to whether they were positive in laboratory and imaging examination: (i) CSF SARS-CoV-2 RT–PCR was positive. This was the first reported case of encephalitis related to the COVID-19 in the world: after the 24-year-old male patient was admitted to the hospital, SARS-CoV-2 RNA was not detected in nasopharyngeal swabs, nor IgM antibodies in serum samples, but SARS-CoV-2 RNA was detected in CSF (Moriguchi *et al*., 2020). Another 40-year-old female patient was hospitalized with encephalitis and found that nasopharyngeal swabs and CSF SARS-CoV-2 RT–PCR were positive (Huang *et al*., 2020b). The SARS-CoV strain was isolated from a previous brain tissue specimen of SARS patients with obvious central nervous system symptoms (Xu *et al*., 2005), which also indirectly proves that the virus can directly invade the nervous system. (ii) No SARS-CoV-2 RNA was found in the CSF, but there was polycythemia, elevated lymphocytes, or elevated protein (Bernard-Valnet *et al*., 2020; Duong *et al*., 2020; Pilotto *et al*., 2020). However, some scholars believe that although SARS-CoV-2 RNA was not detected in CSF, it may be related to the relatively short virus residence or low virus load in the nervous system, or lack of effective detection methods (Duong *et al*., 2020; Ye *et al*., 2020). (iii) There were obvious imaging changes, but all laboratory examinations of CSF were normal. Most of the imaging findings were normal on CT, but abnormal brain parenchymal morphology and signal could be seen on MRI, and fluid-attenuated inversion recovery (FLAIR) and diffusion weighted imaging (DWI) could better display the lesions (Dogan *et al*., 2020; Khoo *et al*., 2020; Wong *et al*., 2020; Ye *et al*., 2020; Zambreanu *et al*., 2020; Zuhorn *et al*., 2021).

The mechanism of COVID-19-associated encephalitis is still unclear. According to these case reports, although there are cases that support the direct invasion of the SARS-CoV-2 into the brain tissue (the virus is found in CSF or brain tissue biopsy), most support the overreaction of nervous system immune cells mainly mediated by autoimmunity/antibodies. Further research is needed to support or refute this hypothesis, which is of significance to the clinical development of a reasonable treatment plan.

### Acute necrotizing encephalopathy (ANE)

Encephalopathy refers to the clinical manifestation of pathology affecting brain function under the action of various factors of extracranial or intracranial origin. Encephalopathy and psychosis are two different mental states. The typical characteristics are disorientation, short-term memory impairment, and inattention in the abnormal awakening state (Perugula and Lippmann, 2016). ANE is a rare para-infectious encephalopathy infected by influenza and other viruses. It mainly occurs in children with influenza, and the typical imaging features are multiple symmetrical edema and necrosis areas in the central nervous system (Kansagra and Gallentine, 2011; Krett *et al*., 2020; Poyiadji *et al*., 2020). However, the reported ANE-related SARS-CoV-2 infections were more common in adults.

The first case of ANE was reported in *Radiology*. A 58-year-old female confirmed with COVID-19 had not only cough and fever, but also mental state changes. Since lumbar puncture was an invasive examination, the patient did not test for SARS-CoV-2 in CSF. But the changes of CT and MRI were consistent with the typical imaging findings of ANE (Poyiadji *et al*., 2020). From the subsequent case reports, it was found that the common mental state changes of ANE related to the COVID-19 were slow response to commands, poor memory, disorientation, and obvious language difficulties, and SARS-CoV-2 was not detected in the cases tested for CSF (Al Mazrouei *et al*., 2020; Farhadian *et al*., 2020; Kihira *et al*., 2020; Krett *et al*., 2020; Umapathi *et al*., 2020).

According to previous studies, ANE caused by SARS-CoV-2 infection was related to intracranial cytokine storm, destroying the blood–brain barrier (BBB), but no virus directly invaded the nervous system (Rossi, 2008). The pathogenesis section next describes how cell storms damage the nervous system. Studies have found that cytokine analysis of CSF showed increased levels of interleukin-1 (IL-1), interleukin-6 (IL-6), interferon gamma (IFN-γ), and tumor necrosis factor alpha (TNF-α), showing that the proinflammatory cytokines were dysregulated. It indirectly proved that cytokine storm was involved in the pathogenesis of ANE (Farhadian *et al*., 2020; Krett *et al*., 2020). However, it is necessary to further study the pathogenesis of COVID-19 related ANE.

### Dysosmia

Dysosmia is one of the most common symptoms in patients with COVID-19 in the acute stage, which is characterized by decreased or loss of olfaction after virus infection. However, at the beginning of the disease outbreak, we did not know enough about this symptom. Subsequent studies found dysosmia may not only be the first symptom before respiratory symptoms (Lechien *et al*., 2020), but also occur in some asymptomatic infected people (Gane *et al*., 2020). The incidence rate of dysosmia reported by various countries was different. A study of 114 COVID-19 patients in France found that 47% of the patients had a dysosmia. Anosmia appeared 4.4 (±1.9) days after infection. The average time of anosmia was 8.9 (± 6.3) days, and 98% of patients recovered within 28 days (Klopfenstein *et al*., 2020). A multicenter study in Belgium found that 357 (85.6%) patients had infection related dysosmia, and 72.6% recovered olfactory function within 8 days after the rehabilitation of the COVID-19 (Lechien *et al*., 2020). However, 11 (5.1%) of 214 confirmed hospitalized patients in Wuhan developed dysosmia, and the authors said that this may be because of incomplete evaluation, which may make this incidence inaccurate (Mao *et al*., 2020). The mechanism of olfactory disorder needs further research.

### Epilepsy

Epilepsy is a disease with complex etiology and it is defined as a kind of neurological disorder caused by excessive discharge of brain neurons from the perspective of physiological mechanism (Scharfman, 2007; Sirven, 2015; Stafstrom and Carmant, 2015). Combined with literature reports, we found that epilepsy in patients with COVID-19 can be divided into two types: first, there is no history of epilepsy and family history. Epileptic symptoms can occur after virus infection, which can be accompanied by cerebrovascular diseases (Sora and Viroj, 2020; Karimi *et al*., 2020; Lu *et al*., 2020; Lyons *et al*., 2020). Second, the patient has a well-controlled history of epilepsy and relapses after infection (Anand *et al*., 2020). Because no virus traces were found in CSF examinations (Anand *et al*., 2020; Lyons *et al*., 2020), the mechanism of epilepsy mainly focuses on two aspects: one is the excessive excitation of neurons induced by cytokine storm, leading to seizures (Libbey and Fujinami, 2011; Singhi, 2011), and the other is the drug side-effects caused by the use of antiviral drugs in hospitalized patients (Ying *et al*., 2021).

## The long-term neuropsychiatric sequelae of COVID-19 survivors

It has been nearly 2 years since the outbreak of the COVID-19. With the continuous increase in the number of infections, the sequelae after the recovery of COVID-19 have been widely reported, but more attention has been paid to the symptoms of the respiratory system and cardiovascular system (Arnold *et al*., 2021; Blanco *et al*., 2021; Trinkmann *et al*., 2021; Wu *et al*., 2021; Xiong *et al*., 2021; Yan *et al*., 2021). According to a previous report, there was still a high incidence rate of neuropsychiatric symptoms in 31 to 50 months after SARS-CoV infection (Lam *et al*., 2009). Therefore, we believe that the world may face a large number of survivors of COVID-19 neuropsychiatric sequelae. Timely understanding of possible symptoms and possible pathogenesis has important socio-economic value in the prevention and treatment of neuropsychiatric sequelae.

### Mental disorder

The outbreak of COVID-19 has had a long-term mental health impact on patients, doctors, and even ordinary people without SARS-CoV-2 infection, including anxiety, depression, post-traumatic stress disorder, and insomnia (Lai *et al*., 2020; Li *et al*., 2020c; Troyer *et al*., 2020). Suicide by emergency doctors also occurred in New York, and some scholars even worry that the COVID-19 pandemic may trigger a wave of suicide (Thakur and Jain, 2020).

At present, there are some differences in the frequency of mental diseases in COVID-19 survivors. Mazza et al (Mazza *et al*., 2021) conducted a prospective study of mental disorder in survivors of the COVID-19. They included 226 COVID-19 survivors and assessed their mental status 1 and 3 months after discharge. The study found that compared with 1 month after discharge, 3 months after discharge the post-traumatic stress disorder, anxiety, and insomnia symptoms of the survivors of the COVID-19 decreased over time, while the depressive symptoms persisted. However, several large cohort studies followed up for 6 months found that the neuropsychiatric sequelae of COVID-19 survivors were mainly sleep difficulties, anxiety, and depression (Al-Aly *et al*., 2021; Huang *et al*., 2021a; Taquet *et al*., 2021a). A recent article published in *The Lancet* shows that the incidence of anxiety or depression at 12 months is slightly higher than that at 6 months, and the proportion of anxiety or depression in women is twice that in men, and age is positively correlated with anxiety or depression (Huang *et al*., 2021b). We think that the reason for this difference may be related to the number of participants included in the study and the length of follow-up.

The pathogenesis of mental disorder in survivors of COVID-19 is unclear whether it is due to direct virus infection or the continuous effect of immune response caused by a cytokine storm (Al-Aly *et al*., 2021; Rogers *et al*., 2020). The previously mentioned 3-month follow-up study found that there was a significant positive correlation between systemic immune-inflammation index (SII  =  platelets × neutrophils/lymphocytes) and depressive symptoms, which may support the role of immune response in the occurrence of mental disorder. The impact of the social environment may not be ignored, such as the reduction of social activities in the recovery stage after discharge or unemployment caused by incomplete recovery of the body.

### Neurodegenerative diseases

Neurodegenerative diseases are a kind of chronic progressive diseases characterized by protein deposition, neuroinflammation, and progressive loss of neurons and axons, mainly including Alzheimer's disease (AD), Parkinson's disease (PD), and multiple sclerosis (MS). There are many causes of neurodegenerative diseases, including neuroinflammation, oxidative stress, aging, and so on (Beltrán-Castillo *et al*., 2018; Singh *et al*., 2019; Tejera *et al*., 2019). Whether the SARS-CoV-2 infection will cause neurodegenerative diseases or accelerate the premature occurrence of neurodegenerative diseases still needs a longer follow-up. Many studies have shown that the neuroinflammatory response is an indispensable key process for the progress of most neurodegenerative diseases (Baik *et al*., 2016; Tejera *et al*., 2019).

AD is one of neurodegenerative diseases caused by neuroinflammatory response, synaptic pruning, and neuron loss (Zhang and Jiang, 2015), and its typical clinical manifestation is cognitive impairment. In view of the various damage that SARS-CoV-2 can cause to the nervous system in the acute phase, its long-term effect on cognitive function is also worthy of attention (Calderón-Garcidueñas *et al*., 2020; Chen *et al*., 2021). A study on the two-way association between COVID-19 and neuropsychiatric diseases showed that the incidence of first diagnosis of AD in people over 65 years old was 1.6% (95% CI 1.2–2.1) in 14 to 90 days after the diagnosis of COVID-19 (Taquet *et al*., 2021b). This study provided preliminary evidence for the link between viral infection and AD. In addition, another study found that the mortality of COVID-19 patients with AD (62.2%) was much higher than COVID-19 patients without AD (26.2%) (Bianchetti *et al*., 2020). So far, the mechanism of AD caused by SARS-CoV-2 infection is still unclear. Some scholars believe that the virus can interact with glutamate and GABAergic may stimulate the excitotoxic reaction caused by the binding of ACE2 receptor in neurons, resulting in brain tissue injury (Guo *et al*., 2020). In addition, viruses invading the nervous system can also widely infiltrate the whole brain through the trans-synaptic transfer, which may be the reason for neurodegenerative changes several months and years after acute infection (Li *et al*., 2020b; Li *et al*., 2012).

PD is a neurodegenerative disease involving the basal ganglia, which is often characterized by motor retardation, static tremor or rigidity. Different from the AD described previously, the mortality of COVID-19 patients with PD (19.7%) was only slightly higher than COVID-19 patients without PD (12.8%) (Fasano *et al*., 2020b), and there was no significant difference in the probability of SARS-CoV-2 infection between patients with PD and the general population (Fasano *et al*., 2020a). Previous studies have shown that patients with PD can show motor and cognitive dysfunction, which may be related to the common pathogenesis of AD and PD (Bezdicek *et al*., 2019; Das *et al*., 2019; Van Bulck *et al*., 2019). Ponsen *et al*. (Ponsen *et al*., 2004) found that anosmia was the early clinical symptoms of PD, while previous studies said that anosmia was often the first symptoms of SARS-CoV-2 infection (Lechien *et al*., 2020). This may be indirect evidence for the interaction between PD and COVID-19 infection. The wide expression of ACE2 receptor in the brain may provide a molecular basis for later neurodegenerative changes (Lukiw *et al*., 2020). Unfortunately, there is no effective clinical evidence to prove these potential mechanisms.

MS is an autoimmune disease, and its pathogenesis is related to neuronal degeneration caused by focal demyelination and inflammation. Since MS patients need to take immunosuppressive drugs, unlike AD or PD, we are no longer concerned with whether MS is a long-term neuropsychiatric sequelae of patients affected by COVID-19, but whether the probability of the patient being infected with SARS-CoV-2 will be increased because of suffering from MS. The current research results on this issue are not uniform. The literature showed that the probability of COVID-19 pneumonia in patients with MS was about 2.5 times higher than that in the general population (Crescenzo *et al*., 2020), but more studies showed that there was no difference between these two groups (Fan *et al*., 2020; Louapre *et al*., 2020). This contradictory result requires more long-term studies to explore whether there is a relationship between SARS-CoV-2 infection and MS.

## Changes of brain structure and functional imaging in long-term follow-up of COVID-19 survivors

### Brain structural changes in COVID-19 survivors at a 3-month follow-up

In a recent study, 51 COVID-19 survivors were followed up for 3 months to study the changes of brain structural. They divided the 51 participants into a mild group (MG, *N* = 19) and a severe group (SG, *N* = 32) based on the severity of their symptoms during the hospitalization period, and respectively compared the images collected by these two groups with the images of 31 healthy controls (HCs). The study showed that the conventional axial T2-FLAIR scans of all COVID-19 survivors showed no abnormalities, but the analysis of structural 3D T1-weighted images (T1WI) and high-resolution diffusion tension images (DTI) found that compared with HC group, the white matter tracts of MG had a few changed, and there was no significant change in gray matter. However, the reduction in cortical thickness and white matter microstructure changes in the SG were more significant than those in the MG, mainly in the frontal lobe and limbic system. These results provide evidence for the nervous system damage of COVID-19 survivors (Qin *et al*., 2021).

### Brain function changes in COVID-19 survivors at a 1-year follow-up

**Figure 1:**
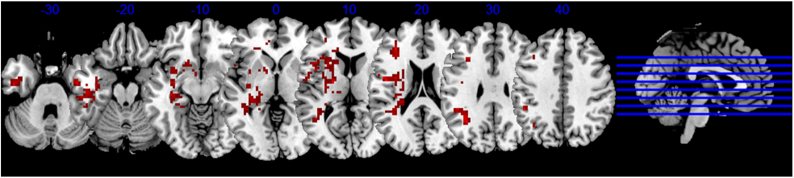
Long-term brain function changes in COVID-19 survivors. The red area indicates the brain area with increased amplitude low-frequency fluctuation value in the COVID-19 rehabilitation patients followed up for 1 year compared with the normal control.

To explore the long-term brain function changes of COVID-19 survivors. Our team recruited 22 COVID-19 rehabilitation patients who were followed up for 1 year and 29 HCs. By comparing the fMRI and clinical data of the two groups, we found that compared with the HC group, the amplitude low-frequency fluctuation (ALFF) value of COVID-19 rehabilitation patients was significantly higher in multiple brain regions, including left anterior central gyrus, hippocampus, caudate, and putamen (Fig. 1). In addition, the increased ALFF value of the left caudate was significantly positively correlated with the score of the ascension insomnia scale, and the ALFF value of the left anterior central gyrus was also positively correlated with the neutrophil count during hospitalization. Our study provides imaging evidence for the long-term neuropsychiatric sequelae of COVID-19 rehabilitation patients, and analyzing the relationship between imaging changes and inflammatory markers may help to reveal the neurobiological mechanism of COVID-19 related neuropsychiatric sequelae (Du *et al*., 2022).

## Potential mechanism of SARS-CoV-2 infection in the nervous system

**Figure 2:**
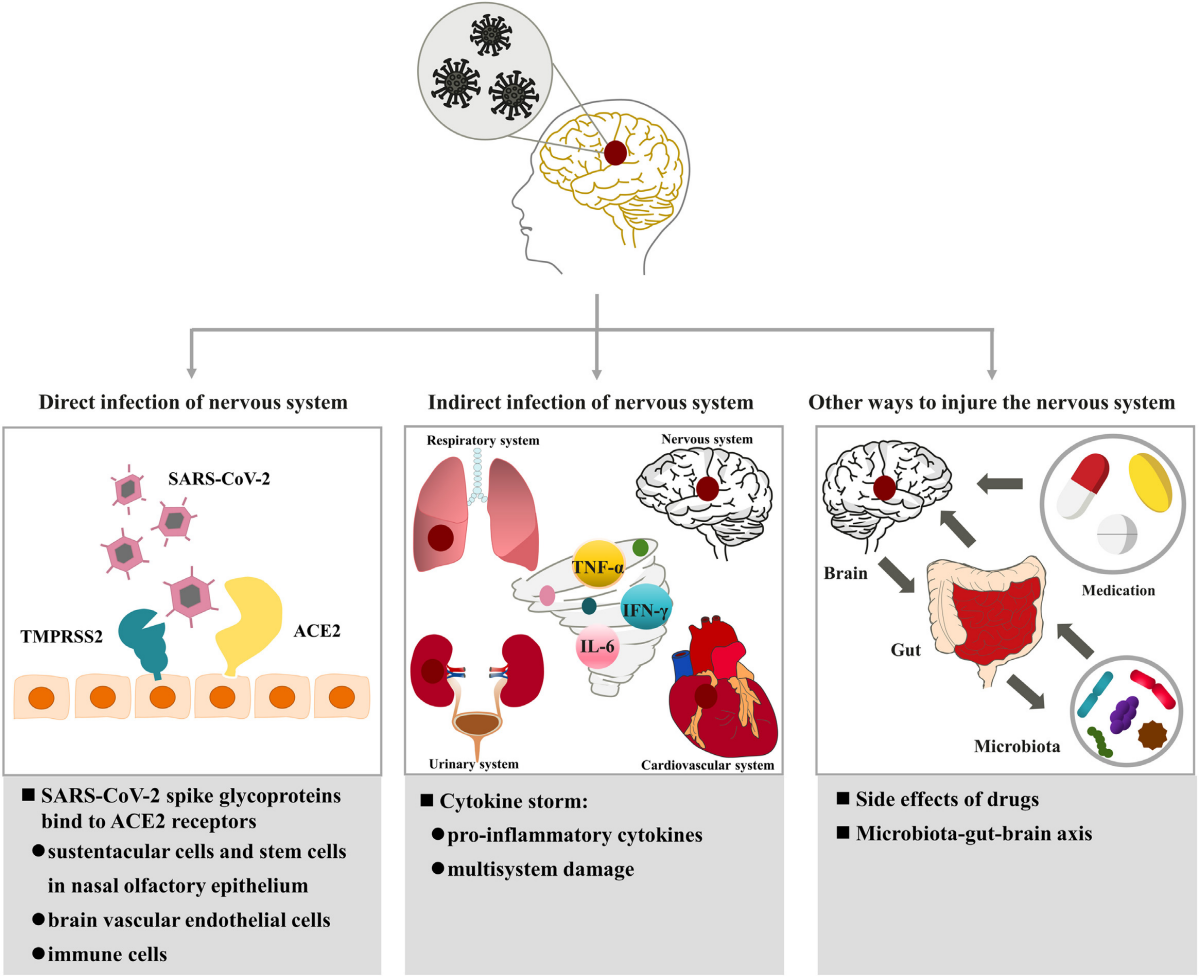
Potential mechanism of SARS-CoV-2 infection in nervous system. TMPRSS2: transmembrane serine protease 2. TNF-α: tumor necrosis factor alpha. IFN-γ: interferon gamma and IL-6: interleukin-6.

With the reports of acute and long-term nervous system symptoms in patients with COVID-19 patients and survivors, some studies have speculated that the symptoms are related to SARS-CoV-2 infection of the nervous system (Achar and Ghosh, 2020; Ellul *et al*., 2020a; Niazkar *et al*., 2020; Troyer *et al*., 2020; Wang *et al*., 2020a). In addition, Zhou *et al*. (Zhou *et al*., 2020a) found that the genetic sequence of the SARS-CoV-2 was 79.6% similar to that of the SARS-CoV, so it may provide indirect evidence to support the symptoms of brain damage caused by SARS-CoV-2 infection. Although the exact pathogenesis of SARS-CoV-2 infection of the nervous system remains unclear, previous studies have suggested some potential mechanisms of SARS-CoV-2 infection in the nervous system (Fig. 2).

### Direct infection of the nervous system

The spike glycoproteins on the surface of SARS-CoV-2 play a vital role in the virus invading the nervous system. Compared to SARS-CoV, the spike glycoproteins on SARS-CoV-2 have a higher affinity for the angiotensin-conversion enzyme 2 (ACE2) receptors (Shang *et al*., 2020; Wang *et al*., 2020c). However, ACE2 receptors exist not only in cardiomyocyte, gastrointestinal epithelial cells, kidney, bladder, and urinary epithelial cells (Zou *et al*., 2020), but also widely in nasal epithelial cells, vascular endothelial cells, brain neurons, astrocytes, and oligodendrocytes (Barrantes, 2020). Previous studies have found that the receptor binding domain of virus was on the S1 subunit of the homotrimeric spike glycoproteins expressed by the SARS-CoV, which provides a basis for the binding of the virus to ACE2 receptor on the cell membrane (Shang *et al*., 2020; Wang *et al*., 2020c; Zhu *et al*., 2013). After they interact, the viral spike glycoproteins are hydrolyzed and cleaved by transmembrane serine protease 2 (TMPRSS2) and lysosomal protease cathepsins (Shang *et al*., 2020). The virus completes the most critical step in crossing the cell membrane (Wang *et al*., 2020c). Similar to the SARS-CoV transmembrane process, SARS-CoV-2 first recognizes cells with low levels of TMPRSS2 through the preactivation of furin (Shang *et al*., 2020), and then also uses TMPRSS2 to cleave and trigger spike glycoproteins (Hoffmann *et al*., 2020). Finally, the SARS-CoV-2 RNA enters healthy cells to replicate. The process by which the virus enters the cell remains to be verified.

Therefore, the SARS-CoV-2 may not only complete the cross synaptic transfer of the virus between neurons and neurons and between neurons and satellite cells directly through exocytosis, endocytosis, and vesicle transport (Li *et al*., 2012), but it may also invade the nervous system through the following three ways in which SARS-CoV-2 spike glycoproteins bind to ACE2 receptors in different cells: (i) SARS-CoV-2 may bind to ACE2 receptors of sustentacular cells (Bilinska *et al*., 2020) and stem cells (Brann *et al*., 2020) in the nasal olfactory epithelium, then transport to the next level neurons through reverse axons (Desforges *et al*., 2019), and finally infect the nervous system. SARS-CoV (McCray *et al*., 2007), MERS-CoV (Jacomy and Talbot, 2003; Li *et al*., 2016), and HCoV-OCR43 (Li *et al*., 2016) have also been reported to invade the brain through olfactory neurons, which indirectly provides evidence for SARS-CoV-2 to enter the brain through the olfactory pathway. (ii) SARS-CoV-2 may bind to ACE2 receptors in vascular endothelial cells (Pezzini and Padovani, 2020), which form the main component of the BBB). BBB refers to the barrier between plasma and brain cells formed by brain capillary wall and glial cells, as well as the barrier between plasma and CSF formed by the choroid plexus (Abbott, 2002; Abbott *et al*., 2006). The BBB can effectively prevent harmful molecules and ions from entering brain tissue from the blood (Abbott, 2002; Abbott *et al*., 2006; Daneman and Prat, 2015; Pardridge, 2005). Electron microscopic images showed that SARS-CoV-2 entered vascular endothelial cells through endocytosis and exocytosis to achieve intercellular transfer of the virus (Paniz-Mondolfi *et al*., 2020). Moreover, SARS-CoV-2 does not replicate in the process of cross endothelial cell transfer, but replicates in the cytoplasm when it reaches its neurons, astrocytes, and oligodendrocytes (Baig *et al*., 2020). (iii) SARS-CoV-2 may bind to ACE2 receptors in immune cells (Desforges *et al*., 2014). SARS-CoV-2 may use lymphocytes, granulocytes, and monocytes involved in immune responses to pass through the BBB. Recently, a new hypothesis has been proposed that when the body is infected by the SARS-CoV, macrophages not only play a defensive role, but also use the “Trojan horse” mechanism to help the virus enter the nervous system (Abassi *et al*., 2020). In other words, after the immune response caused by virus infection, macrophages carry the virus through the BBB and specifically anchor in the brain parenchyma. ACE2 receptors on the surface of sustentacular cells and stem cells in nasal olfactory epithelium, vascular endothelial cells of the BBB, and immune cells provide binding sites for the SARS-CoV-2 to directly invade the nervous system.

### Indirect infection of the nervous system

Cytokine storm refers to an uncontrolled excessive inflammatory reaction, which starts locally and spreads further in the body through systemic circulation, which may be the mechanism by which the virus indirectly infects the nervous system (Jose and Manuel, 2020). When the human immune system cannot produce an appropriate adaptive immune response to SARS-CoV-2, the persistence of the virus and the extension and amplification of the inherent immune mechanism lead to the dysfunction of immune response, resulting in the high inflammatory state of cytokine storm (Pelaia *et al*., 2020). Cytokine storm has also been observed in patients infected with SARS-CoV and MERS-CoV (Mahallawi *et al*., 2018; Wong *et al*., 2004). Like SARS-CoV and MERS-CoV, patients infected with SARS-CoV-2 may have elevated levels of proinflammatory cytokines, such as TNF-α, IFN-γ, and IL-6 (Han *et al*., 2020; Huang *et al*., 2020a). In particular, there are only elevated levels of antiinflammatory cytokines in COVID-9 patients, including IL-2, IL-4, and IL-10 (Benameur *et al*., 2020). This may be because IL-6 can down regulate intraendothelial adhesion and tight junction proteins and increase the paracellular permeability of microvascular endothelial cells, which may affect the integrity of BBB (Rochfort *et al*., 2014). Therefore, the cytokine storm caused by SARS-CoV-2 may damage the nervous system from two aspects. On the one hand, it increases the microvascular permeability of the nervous system, so that the virus can enter the brain through the BBB (Daneman and Prat, 2015; Pardridge, 2005). A cytokine storm can also activate the coagulation system and lead to the formation of cerebral microthrombosis (Benameur *et al*., 2020; Ehrlich *et al*., 1998). Therefore, SARS-CoV-2 may indirectly damage the nervous system through the cytokine storm.

### Other ways to injure the nervous system

Besides the mechanisms mentioned previously, the side-effects of therapeutic drugs and the microbiota–gut–brain axis may also be the potential mechanisms causing neurological symptoms, but they need to be further investigated by further research.

Severe patients and patients in the acute phase of COVID-19 were often treated with corticosteroids (Liu *et al*., 2020; Mehta *et al*., 2020; Mo *et al*., 2020; Wan *et al*., 2020), and a number of studies have shown that 35% of patients treated with corticosteroids have internal neuropsychiatric symptoms such as cognitive and sleep disorders, mania, and depression (Brown and Chandler, 2001; Warrington and Bostwick, 2006), which indicates that corticosteroid treatment may mediate nervous system damage in patients with COVID-19 (Troyer *et al*., 2020).

The concept of the microbiota–gut–brain axis was proposed in 2009 (Rhee *et al*., 2009). The gut microbiota may affect the nervous system through the microbiota–gut–brain axis in regulating anxiety, emotion, cognition, and pain (Cryan and Dinan, 2012). An animal experiment showed that mice infected with *Trichuris muris* showed obvious anxiety-like behaviors, and cognitive dysfunction-like behaviors were observed 30 days after the symptoms of the infection subsided (Bercik *et al*., 2010). Therefore, based on previous studies, we suspect that changes in the gut microbiota of patients with COVID-19 may cause neuropsychiatric symptoms through regulating the microbiota–gut–brain axis.

## Summary and prospect

With the increasing number of COVID-19 cases worldwide, more and more evidence has showed that neurological symptoms are related to viral infections, but the exact pathogenesis is still unclear. We believe that imaging examinations have advantages in showing acute damage to the nervous system and long-term neuropsychiatric sequelae. In the acute phase of virus infection, conventional sequences such as T2-FLAIR and DWI can suggest that patients with COVID-19 are complicated with acute ischemic stroke or encephalitis when other tests are negative. However, nontraditional MRI sequences, especially 3D T1WI, DTI, and fMRI, may be more meaningful to show long-term changes in brain structure and function. These long-term follow-up changes in structure and function may also help us understand the causes of neuropsychiatric symptoms in COVID-19 survivors. The underlying mechanisms associated with SARS-CoV-2 infection are diverse and unclear. We believe that the research on the mechanism of infection should not only study the way of invading the nervous system, but should also pay attention to the underlying diseases that may increase the susceptibility of this group of people. This may require more research to explore the pathogenesis of such comorbidities.

Our present paper, therefore, mainly focused on the damage to the nervous system, the acute and long-term neuropsychiatric sequelae, and the potential mechanism of SARS-CoV-2 infection. In light of the continuous attention to neuropsychiatric sequelae and current understanding about the long-term damage of the virus to the nervous system, formulating effective sequelae prediction and prevention is of great significance, and thus can provide important clues for the nervous system damage of future public health crises.
